# Pigment signatures of algal communities and their implications for glacier surface darkening

**DOI:** 10.1038/s41598-022-22271-4

**Published:** 2022-10-21

**Authors:** Laura Halbach, Lou-Anne Chevrollier, Eva L. Doting, Joseph M. Cook, Marie B. Jensen, Liane G. Benning, James A. Bradley, Martin Hansen, Lars C. Lund-Hansen, Stiig Markager, Brian K. Sorrell, Martyn Tranter, Christopher B. Trivedi, Matthias Winkel, Alexandre M. Anesio

**Affiliations:** 1 Department of Environmental Science, Aarhus University, iClimate, Roskilde, Denmark; 2 GFZ German Research Centre for Geosciences, Potsdam, Germany; 3 Department of Earth Sciences, Freie Universität Berlin, Berlin, Germany; 4 School of Geography, Queen Mary University of London, London, United Kingdom; 5 Department of Biology, Aarhus University, Aarhus, Denmark; 6 Department of Ecoscience, Aarhus University, Roskilde, Denmark

**Keywords:** Water microbiology, Microbial ecology, Freshwater ecology

## Abstract

Blooms of pigmented algae darken the surface of glaciers and ice sheets, thereby enhancing solar energy absorption and amplifying ice and snow melt. The impacts of algal pigment and community composition on surface darkening are still poorly understood. Here, we characterise glacier ice and snow algal pigment signatures on snow and bare ice surfaces and study their role in photophysiology and energy absorption on three glaciers in Southeast Greenland. Purpurogallin and astaxanthin esters dominated the glacier ice and snow algal pigment pools (mass ratios to chlorophyll *a* of 32 and 56, respectively). Algal biomass and pigments impacted chromophoric dissolved organic matter concentrations. Despite the effective absorption of astaxanthin esters at wavelengths where incoming irradiance peaks, the cellular energy absorption of snow algae was 95% lower than anticipated from their pigmentation, due to pigment packaging. The energy absorption of glacier ice algae was consequently ~ 5 × higher. On bare ice, snow algae may have locally contributed up to 13% to total biological radiative forcing, despite contributing 44% to total biomass. Our results give new insights into the impact of algal community composition on bare ice energy absorption and biomass accumulation during snow melt.

## Introduction

Algal communities thrive on glacier and ice sheet surfaces around the world^1,2^. They contribute to regional nutrient and carbon cycling^3–5^ and significantly reduce surface albedo, accelerating the melting of ice^6–9^ and snow^10–13^. Their most striking adaptation is their dark pigmentation, making them effective absorbers of solar irradiance^6,10,14–16^. They account on average for 9–13% of surface ice melt on the south-western margin of the Greenland Ice Sheet (GrIS)^6^ and 13% of snow albedo reduction across the Arctic^10^.

The algal community on bare ice is typically dominated by glacier ice algae^1,2^ with the filamentous *Ancylonema nordenskiöldii* (Zygnematales, Chlorophyceae) and the mostly unicellular *A. alaskanum* (formerly *Mesotaenium berggrennii*^17^). Glacier ice algae accumulate large amounts of secondary, brown-coloured phenolic pigments, mainly purpurogallin-derivatives, in vacuoles in their cells^17,18^. Purpurogallin-derivatives absorb light broadly across the near-UV and visible wavelength range (λ_max_ 338 nm^18,19^) and influence the absorption properties of the glacier ice algal cells^15,16^. The accumulation of secondary pigments protects the internal photosynthetic apparatus from potential damage by excess irradiance^15,18,20^ and can also be observed in the unicellular snow algae (mostly Chlamydomonadales, Chlorophyta)^1^. They transition from green-coloured, motile cells to red-coloured non-motile, cysts, rich in carotenoids, as the snowpack ripens and melts during the spring thaw^21–23^. These carotenoids are stored in lipid globules and consist mainly of astaxanthin isomers and their fatty-acid ester derivatives, hereafter referred to as astaxanthin esters. These absorb solar irradiance in both the visible (λ_max_ 470 nm, all-*trans-*astaxanthin) and the UV range (368 nm, 13Z-*cis-*astaxanthin)^20,24,25^. Exposure to UV radiation and nitrogen limitation are major factors triggering the production of photoprotective carotenoids in snow algae^21^. In addition, it has been postulated that secondary pigments may also help to generate meltwater around the cells by dissipating heat from absorbed sunlight^15,26^. Since the purpurogallin-derivatives and astaxanthin esters are free of nitrogen and phosphorus, they can be produced even during periods of low nitrogen and phosphorous availability on bare ice^18^ or snow^21^.

Algal pigments and other organic substances can enter the pool of coloured dissolved organic matter (CDOM) in the environment upon excretion by the algae or leakage from dead cells^27,28^. It has been demonstrated that CDOM can be of ecological relevance by attenuating UV light around algal cells in other aquatic environments^27,29,30^, but no data are available yet from supraglacial bare ice environments.

The contribution of pigmented algae to the melting of the GrIS is not yet fully understood^15,31^. Uncertainties on the light absorption efficiencies of glacier ice^15^ and snow algae^24,31^ have been highlighted as one of the major factors limiting our ability to assess algal-driven surface darkening^31^. Since the pigmentation of algae influences their light absorption and, hence, their darkening potential^15,16^, it is crucial to improve our understanding of the extent of pigment accumulation in glacier ice and snow algae for deciphering their impact on surface darkening. For glacier ice algae, in particular, data on their pigmentation are scarce and only available from the south-western margin of the GrIS^15,19^ and it remains unclear to what extent their pigmentation could vary regionally. Furthermore, snow and glacier ice algae are traditionally studied independently, however, community mixing occurs upon melting of seasonal snowpacks, so that snow algae are being washed onto the bare ice where they remain on its surface^32^. This community mixing on the bare ice has so far been rarely considered^32^ and could impact glacier surface darkening and snow algal population survival since their cysts can act as inoculum for the next melting season^33,34^.

Here, we studied the relative abundance, pigmentation, and photophysiology of glacier ice and snow algae along with snow and ice CDOM concentrations on three glaciers on SE-Greenland. The different sampling locations enable us to compare algal communities and their adaptation strategies, which may be closely linked to the different environmental conditions of their respective habitats. We analyse how snow and glacier ice algal pigment signatures manifest in their individual light-absorption properties by combining estimated (pigment-derived) and empirically measured (including pigment packaging effects in algal cells) absorption properties. The absorption coefficients were subsequently used to estimate snow and glacier ice algal contribution to biological radiative forcing on the three studied glaciers.

## Methods

### Field site description and sample collection

**Figure 1:**
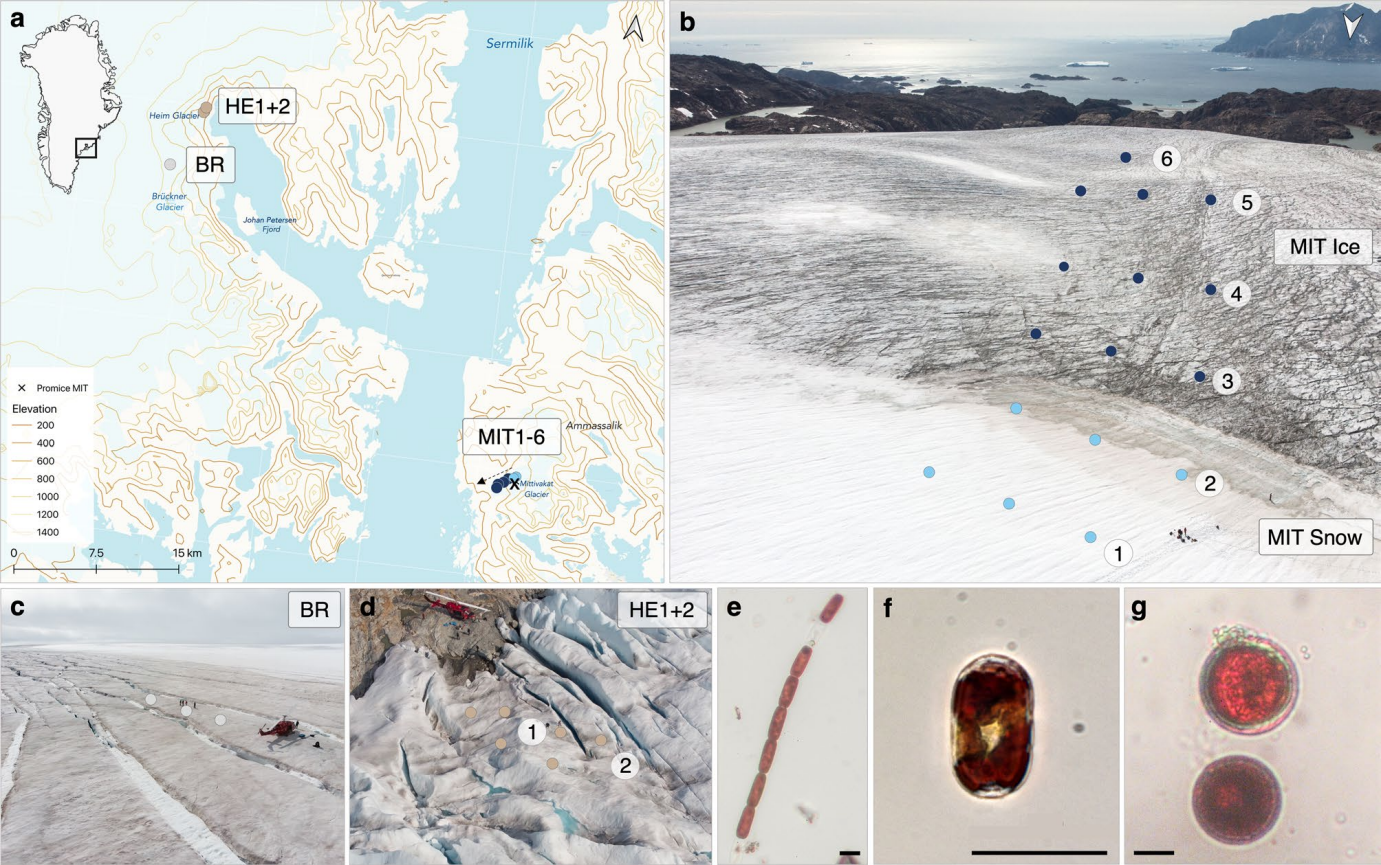
Algal community sampling locations in SE-Greenland: (**a**) Black square on the Greenland map insert marks the sampling region; sampling locations are shown by dots on the topographic map (Mittivakkat = MIT, Bruckner = BR, Heim = HE) and ice coverage is indicated by the bright blue area on land. The map was created using QGIS v.3.16 (http://www.qgis.org)^78^. (**b–d**) Drone photos of sampling locations on Mittivakkat, Bruckner and Heim Glaciers. On Mittivakkat, sampling was done along a transect of 1.14 km length along a snow-to-ice gradient with decreasing altitude. Snow and bare ice sampling sites are grouped for habitat and glacier comparisons as “MIT Snow” and “MIT Ice”. (**e–g**) The three dominant algal species across glaciers: glacier ice algae (*A. nordenskiöldii*, *A. alaskanum*) and snow algae (Chlamydomonadales sp.) with scale bars of 20 µm.

Snow and ice samplings were performed on Mittivakkat (MIT), Bruckner Glacier (BR) and Heim Glacier (HE) in SE-Greenland (65°41′40″N, 37°50′05″W), between 20 and 27 July 2019 (Fig. 1; site details and environmental parameters are shown in Supplementary Table S1and S2).

On Mittivakkat, a land terminating glacier of the Ammassalik island ice field, triplicate samples were collected along a west–sloping transect extending from above to below a persistent snowpack (Fig. 1b; MIT1-6; 451–274 m altitude). Additional samples were collected on two glaciers located across the Sermilik fjord, which are part of the GrIS and are marine-terminating into the Johan Petersen Fjord: Bruckner Glacier (BR; Fig. 1c; 65°59′35’’N, 38°26′50’’W) and Heim Glacier (HE1 + 2; Fig. 1d; 65°57′1’’N, 38°31′8’’W). On Bruckner Glacier, samples were collected on a plateau located at 942 m, where a thin layer of snow was still present but had a surface melt-freeze crust, while on Heim Glacier, samples were collected at 286 m altitude at the glacier margin on bare ice. At all sampling sites, at least three samples were collected with a minimum spacing of ∼10 m between them, which are presented as averages per site. For each sample, the upper ∼2 cm of the ice/snow surface was scraped off and collected into sterile Whirl-pack bags (Nasco, USA) for cell counts, Pulse-Amplitude-Modulated (PAM)-fluorometry, pigments and algal absorption analyses or into Nalgene bottles, which were pre-rinsed with sample water, for CDOM analysis. After melting the samples slowly in the dark for approx. 24 h, sub-samples were taken for the different analyses of which the PAM measurements were carried out directly in the field.

### Cell counts, biovolume and biomass estimations

To quantify the relative abundance of snow (Chlamydomonadales sp.) and glacier ice algae (*A. nordenskiöldii* and *A. alaskanum*), sub-samples of 2 ml were preserved with glutaraldehyde (2% final concentration) and stored at 4 °C in the dark. Cells were counted by performing 3–6 replicate counts of a full haemocytometer chamber per sample (3.2 µl; Fuchs-Rosenthal, Lancing, UK) using an inverted light microscope (Olympus CK2). Photos of randomly selected cells were taken and cell lengths and widths were measured using ImageJ (Version 1.52p; National Institutes of Health, Bethesda, MD, USA). Cell volumes (μm^3^) were calculated using cylindrical shapes for glacier ice algae and spherical shapes for snow algae after Hillebrand et al.^35^. The cellular biovolumes were converted to the carbon equivalent biomass (pg C cell^−1^) using the biovolume to carbon mass conversion constants (0.109 and 0.991) from Montagnes et al.^36^ to obtain the total mass of carbon of the algal community per snow or ice meltwater volume (µg C ml^−1^).

### Photophysiology

To explore the link between algal relative abundances and pigmentation with photophysiology, we assessed the in vivo efficiency of energy conversion of Photosystem II (PSII, i.e., photosynthetic performance) of the algal communities by measuring variable chlorophyll-fluorescence directly in the field. This was done by using a Phyto-PAM (Walz GmbH, Germany) fitted with a Phyto-ED detector unit equipped with a blue light detector system (470 nm)^37^. From each sample, the maximum quantum efficiency (F_v_/F_m_) was measured after at least 30 min of dark acclimation at ~ 5 °C. During rapid light curves, the effective quantum yield of PSII (Y(II)) as a function of increasing light intensities was measured with the pulsed light technique and subsequently converted to relative electron transfer rates (rETR). The photosynthesis-irradiance (PI) model of^38^ was fitted to the rETR vs. PAR data using the phytotools package^39^ in R. Instrument settings and calculations can be found in Supplementary Methods.

### Pigment sample processing, extraction, and analysis

We quantified major hydrophilic and lipophilic pigments of the algal community. Depending on the biomass 50–300 ml of each sample were filtered onto Whatman GF/F filters (nominal pore size 0.7 µm) in the field. These were then wrapped in aluminium foil and stored in a cryoshipper for transport to the home laboratory. There, filters were freeze-dried (− 110 °C) and a two-phase extraction was used to quantify major lipophilic and hydrophilic pigments following Aigner et al. and Holzinger et al.^40,41^ with some modifications as described in Supplementary Methods. Lipophilic pigments were analysed on a high-pressure liquid chromatography system (HPLC; Diode array detector, Shimadzu) and quantified using analytical standards (Supplementary Methods). The peak at 338 nm was associated with purpurogallin extracted from algal cells, which was confirmed by additional absorption tests (Supplementary Fig. S1). The water-soluble purpurogallin was quantified by measuring the absorption of the hydrophilic extract after dilution with solvent at 338 nm and in triplicates on a microvolume spectrophotometer (220–750 nm, 1 nm intervals, Nanodrop 200/2000c, Thermofisher, Germany) that was calibrated against a purpurogallin standard (CarboSynth, UK). The two main purpurogallin derivatives (glycolised and unglycolised) were further identified using an ultra-high performance liquid chromatography system hyphenated with a nano-liquid chromatography Orbitrap high-resolution tandem mass spectrometer (LC-HRMS/MS) (Supplementary Methods and Fig. S2). Pigment data are presented in mM concentrations to better compare pigment groups of which not the whole molecule acts as chromophore absorbing light.

### Coloured dissolved organic matter (CDOM) absorbance

Subsamples of melted ice and snow were filtered through 0.2 µm PES filters into combusted (450 °C) 60 ml amber glass vials, transported back to the laboratory and then stored at 4 °C with short exposure to elevated room temperatures. Elevated storage temperature effect on sample CDOM quality quantity was assessed by comparing features with acidified CDOM samples, showing only effects on a_375_ within 10%. Prior to analysis, CDOM samples were acclimated to room temperature and measured on a Shimadzu UV 2700 dual beam spectrophotometer using the 10 cm cuvette set-up at an interval of 3 nm. All measurement blanks were prepared using fresh Milli-Q (18.2 ΜΩ cm). The absorption coefficient *a*_*(*λ)_ was calculated with the factor 2.303 being the natural logarithm of 10, $OD$_λ_ representing the optical density and *l* being the cuvette pathlength (m^−1^):

1 $$a (\lambda )=2.303\times \frac{OD\left(\lambda \right)}{l}$$

A baseline correction was done relative to the mean absorption values at 750 nm. CDOM absorptions were normalised to dissolved organic carbon concentration (mean of 0.996 mg L^−1^; Supplementary Methods) to calculate the mass-specific absorption coefficient.

### Algal absorption and pigment packaging

We assessed glacier ice and snow algal absorption using (i) reconstructed “unpackaged” absorption coefficients, which are particularly useful to study the role of individual pigment groups for the total algal absorption, and (ii) “packaged” cellular absorption coefficients. For the first approach, following Bidigare et al.^42^, we reconstructed algal absorption coefficients ($a$; m^−1^) based on their pigment composition according to Eq. (2):

2 $$a (\lambda )=\sum ai\times \mathrm{Ci}$$

where $a$_i_ is the molar mass-specific absorption coefficient of a specific pigment (m^2^ mM^−1^, Supplementary Methods and Fig. S3) and c_i_ is the average concentration of the pigment (mM) in glacier ice and snow algae, respectively. This approach assumes that algal absorption is a linear combination of the absorption of each pigment^42^. The molar absorption coefficient for purpurogallin was derived by dividing the absorbance (m^−1^) of the hydrophilic extracts by the given concentration of the standard in the cuvette (mM m^−3^). The coefficient for all-*trans*-astaxanthin was taken from Clementson and Wojtasiewicz^43^ and was corrected for absorption differences of the *cis*-isomer. Due to the lack of astaxanthin ester standards, the unesterified astaxanthin coefficients were conservatively corrected for molar mass differences using the astaxanthin-ester composition of red snow algal cysts in Bidigare et al.^24^. All other absorption coefficients were taken from Bidigare et al.^44^. In the second approach, we measured the cellular absorption coefficients of algal cells directly. In the field, we filtered algal cells from the snow (MIT2) and the site with the highest glacier ice algal relative abundance (HE1) onto GF/F filters, which were stored in plastic dishes for ~ 24 h at room temperature and then in darkness at − 20 °C until analysis. Optical densities (OD_s_) of the filters were measured on a Shimadzu UV 2700 dual-beam spectrophotometer equipped with an integrating sphere (2600-ISR). A wetted GF/F filter served as blank to account for scattering and absorption of the filter itself. Algal absorption spectra were corrected for the pathlength amplification effect (ß) according to Bricaud and Stramski^45^ and the optical density of the cells on the filters (OD_s (λ)_) was converted to the optical density of particles in suspension (OD_sus (λ)_):

3 $${OD}_{sus}\left(\lambda \right)=0.423\times {OD}_{s}\left(\lambda \right)+0.479\times {{OD}_{s}\left(\lambda \right)}^{2}$$

Then, OD_sus (λ)_ was converted to the absorption coefficient (a_(λ)_; Eq. (1)) using the hypothetical path length *l* (m^−1^) derived by the ratio between filtered sample volume and sample area on the filter. Algal absorption coefficients (m^−1^) were subsequently normalised to cell concentrations per meltwater volume (cells m^−3^), total algal biovolume per meltwater volume (µm^3^ m^−3^) and chl *a* concentrations per meltwater volume (mM m^−3^). Since glacier ice algae cells are less robust than snow algal cysts and purpurogallin is hydrophilic, the absorption spectrum of glacier ice algae (Supplementary Fig. S4) could be biased by pigment leakage after thawing of the filters. Therefore, we used the literature spectra of glacier ice algal absorption including packaging from Williamson et al.^15^, also used in Cook et al.^6^.

### Radiative forcing

We estimated instantaneous radiative forcing (IRF), which is defined as the energy reemitted by particles as heat to their surroundings. Therefore, we calculated the total algal absorption of glacier ice algae and snow algal populations (m^2^ ml^−1^) by multiplying their biovolume-specific absorption coefficients (m^2^ µm^−3^) with the total algal biovolume per meltwater (µm^3^ ml^−1^) for each sampling region. The total algal absorption was subsequently multiplied with the incoming spectral irradiance (midday 12 pm; W m^−2^ _*λ300−750 nm*_ at 1 nm resolution) obtained from the PVSystems solar irradiance program (https://pvlighthouse.com.au*,* last access: January 2022) for 65^°^N and 24 July 2019 (Supplementary Fig. S5), yielding the algal-driven IRF in W ml^−1^. Following previous studies^6,15,26^, we chose the default settings of the model, i.e., the global irradiance (being the sum of direct and diffuse irradiance) and horizontal plane (perpendicular to the direction of sunlight). We do not integrate the IRF throughout the day to compare the impact of the different algal cell types and their varying biomass only. The sum of the wavelength-specific IRF yields the total energy generated by algae that is available to melt proximal frozen water. The energy used for photosynthesis (~ 1.7%) was subtracted from the IRF for each scenario. We disregarded the effect of scattering by algal cells following Williamson et al.^15^.

### Data analysis and statistics

All data analysis were performed in RStudio v.1.1.414 (RStudio, Inc 2018). To test for significant differences between sites we performed a one-way analysis of variance (ANOVA) and in case of a significant result (using p < 0.05), a Tukey Honest Significant Differences post hoc test was performed. Assumptions of normality and homoscedasticity of residuals were tested using Shapiro–Wilk normality tests and Levene’s test. Data were transformed if the assumptions were not met (log-transformation for count data and sqrt-transformation for right-skewed data). For correlations tests, the Spearman correlation coefficient was generally used and the Pearson correlation coefficient for log-transformed data.

## Results

### Algal abundance, biomass, and community composition across glaciers

**Figure 2:**
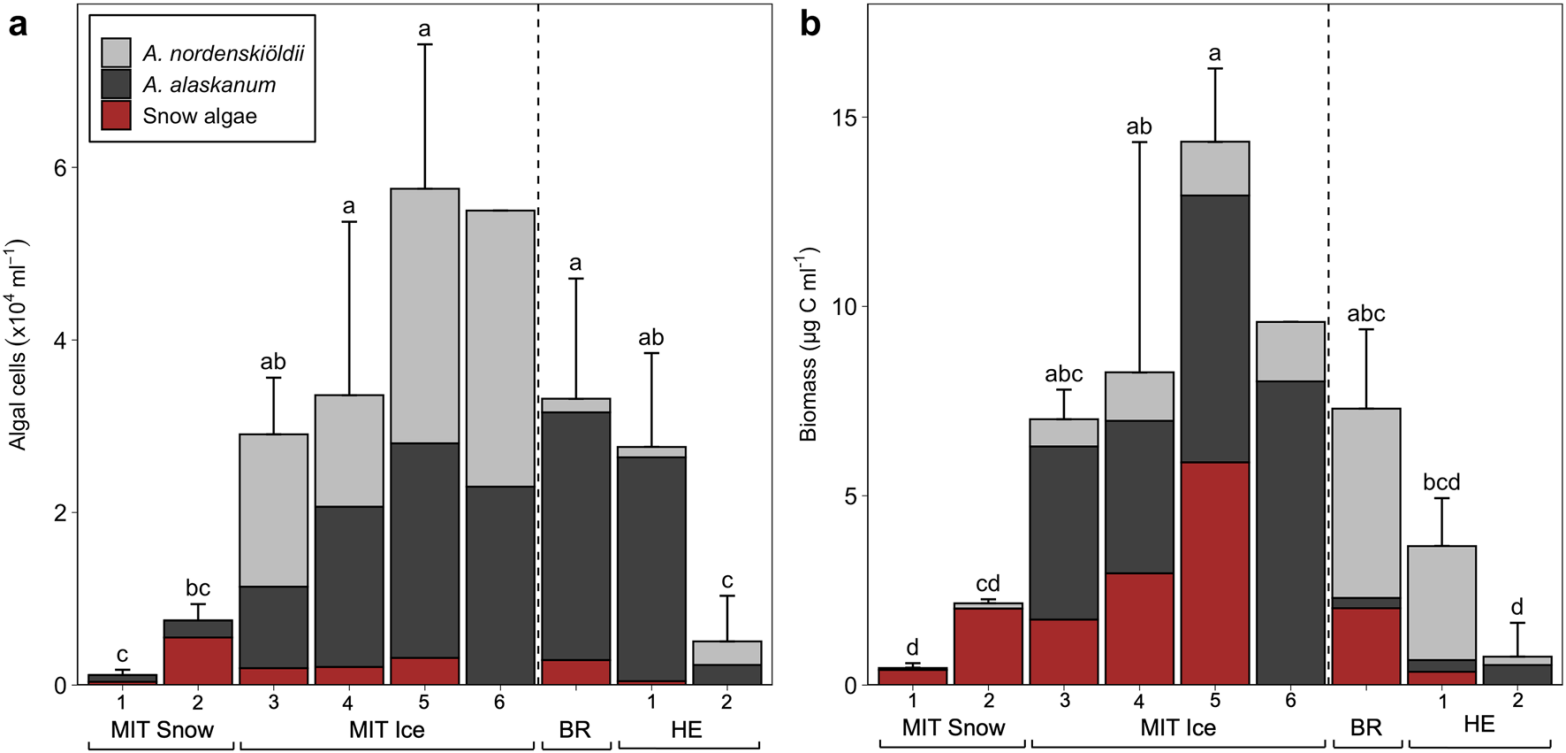
Algal abundance (**a**) and biomass (**b**) displayed as averages per sampling site (MIT = Mittivakkat, BR = Bruckner Glacier, HE = Heim Glacier). Coloured bars indicate the algal group contributions to overall total abundance and biomass with corresponding standard deviation bars on top. Colours indicate relative contributions by glacier ice algae (*A. nordenskiöldii* and *A. alaskanum*) and red snow algae (Chlamydomonadales sp.). Unequal lowercase letters indicate significant differences between sites.

We sampled replicated snow and bare ice-dominated sites on three glaciers (Fig. 1) and studied algal relative abundances and biomass (Fig. 2; biovolumes and algal concentrations are shown in Supplementary Table S4). On the snow sites on Mittivakkat (MIT1 + 2), snow algae (resembling *Sanguina nivaloides*) had an average cell volume of 3963 ± 675 µm^3^ and cell numbers ranged from 0.04 to 0.55 × 10^4^ cells ml^−1^, contributing on average 74% to the community biomass (1.2 ± 0.9 µg C ml^−1^). The bare ice algal community on Mittivakkat (MIT3-6) was dominated by glacier ice algae, *A. nordenskiöeldii* and *A. alaskanum*, in almost equal proportions of both species with average cell volumes of 2307 ± 421 and 822 ± 133 µm^3^, respectively. The total glacier ice algal abundance ranged from 1.9 to 7.5 × 10^4 ^cells ml^−1^ and their biomass from 1.71 to 12.97 µg C ml^−1^. Overall, total algal abundance increased with decreasing altitude along the transect: glacier ice algae on the bare ice by a factor of 2 (2.7 to 5.5 × 10^4^ cells ml^−1^ from 415 to 274 m), and snow algae on the snow sites by a factor of 15 (0.03 to 0.5 × 10^4^ cells ml^−1^ from 451 to 445 m). Notably, snow algal cell volumes increased from 7130 to 20,101 µm^3^ on the bare ice transect (except MIT6 where snow algae were absent), so snow algae still made up on average 44% of algal biomass on the bare ice. On Heim Glacier, the average glacier ice algal abundance was lower than on the bare ice sites on Mittivakkat (on average 1.6 × 10^4^ ± 1.4 × 10^4^ compared to 4.5 × 10^4^ ± 1.9 × 10^4 ^cells ml^−1^) and snow algae contributed only up to 9% to the community biomass*.* Even though snow was still present on Bruckner Glacier, glacier ice algae dominated the algal community with numbers ranging from 1.5 to 3.0 × 10^4^ cells ml^−1^ representing on average 72% of the community biomass. Snow algal abundance only ranged between 0.02 and 0.4 × 10^4^ cells ml^−1^. While *A. alaskanum* was the dominant glacier ice algal species on Heim Glacier and Bruckner Glacier, *A. nordenskiöldii* was dominant on Mittivakkat.

### Community photophysiology

**Figure 3:**
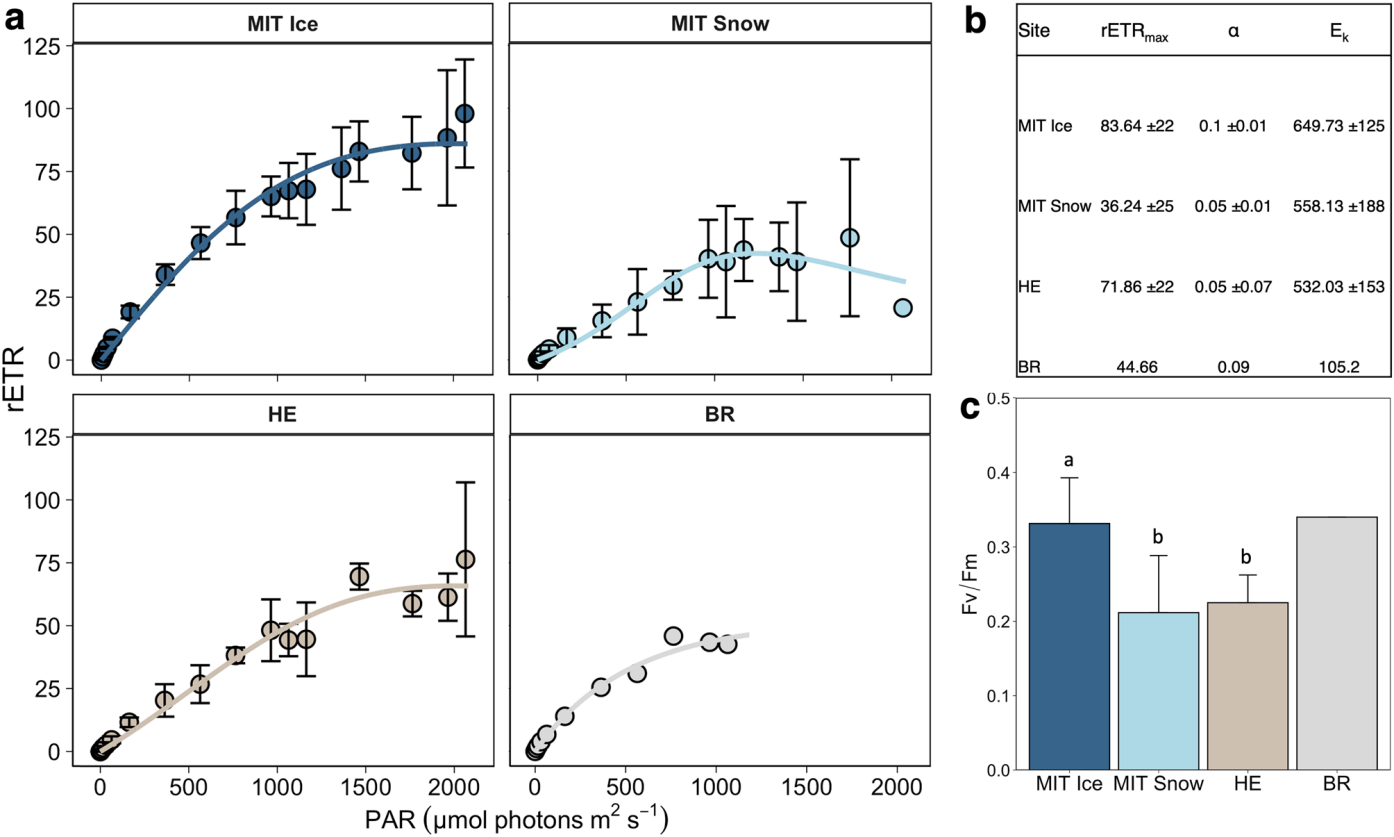
Phyto-PAM derived photophysiological parameters of the algal community: (**a**) mean relative electron transfer rate (rETR) as a function of PAR during rapid light curves with error bars (standard deviation). (**b**) Parameters derived from rapid light curves: maximum relative electron transfer rate (rETR_max_), light utilisation efficiency (α) and the light saturation coefficient (E_k_). (**c**) Maximum quantum efficiency (F_v_/F_m_) of dark-acclimated samples with differing letters indicating significant differences between regions.

We found substantial differences in the photophysiology between snow and ice communities on Mittivakkat; the photoadaptation (F_v_/F_m_; 0.22 ± 0.08 vs. 0.35 ± 0.06, p < 0.001, n = 12) and photosynthetic activity (rETR_max_; 36 ± 25 vs. 84 ± 22 µmol é m^−2^ s^−1^, p < 0.05, n = 12) were lower in the community collected from the snow compared to the bare ice (Fig. 3a,c). There was also a lower efficiency in light utilisation (α; 0.05 ± 0.01 and 0.1 ± 0.01) and a lower light saturation coefficient (E_k_; 558 ± 188 and 649 ± 125 µmol PAR m^−2^ s^−1^) in the snow community compared to the bare ice (Fig. 3b). On Bruckner Glacier (refrozen snow), the rETR_max_ and E_k_ were also lower than on Heim Glacier (bare ice): rETR_max_ 44 vs. 72 ± 22 µmol é m^−2^ s^−1^; E_k_ 105 vs. 532 ± 153 µmol PAR m^−2^ s^−1^. Moreover, F_v_/F_m_ of the bare ice community on Mittivakkat was significantly higher than that of the Heim Glacier community (0.35 ± 0.06 vs. 0.22 ± 0.03, respectively, p < 0.05, n = 12; Fig. 3c).

### Pigment signatures

**Figure 4:**
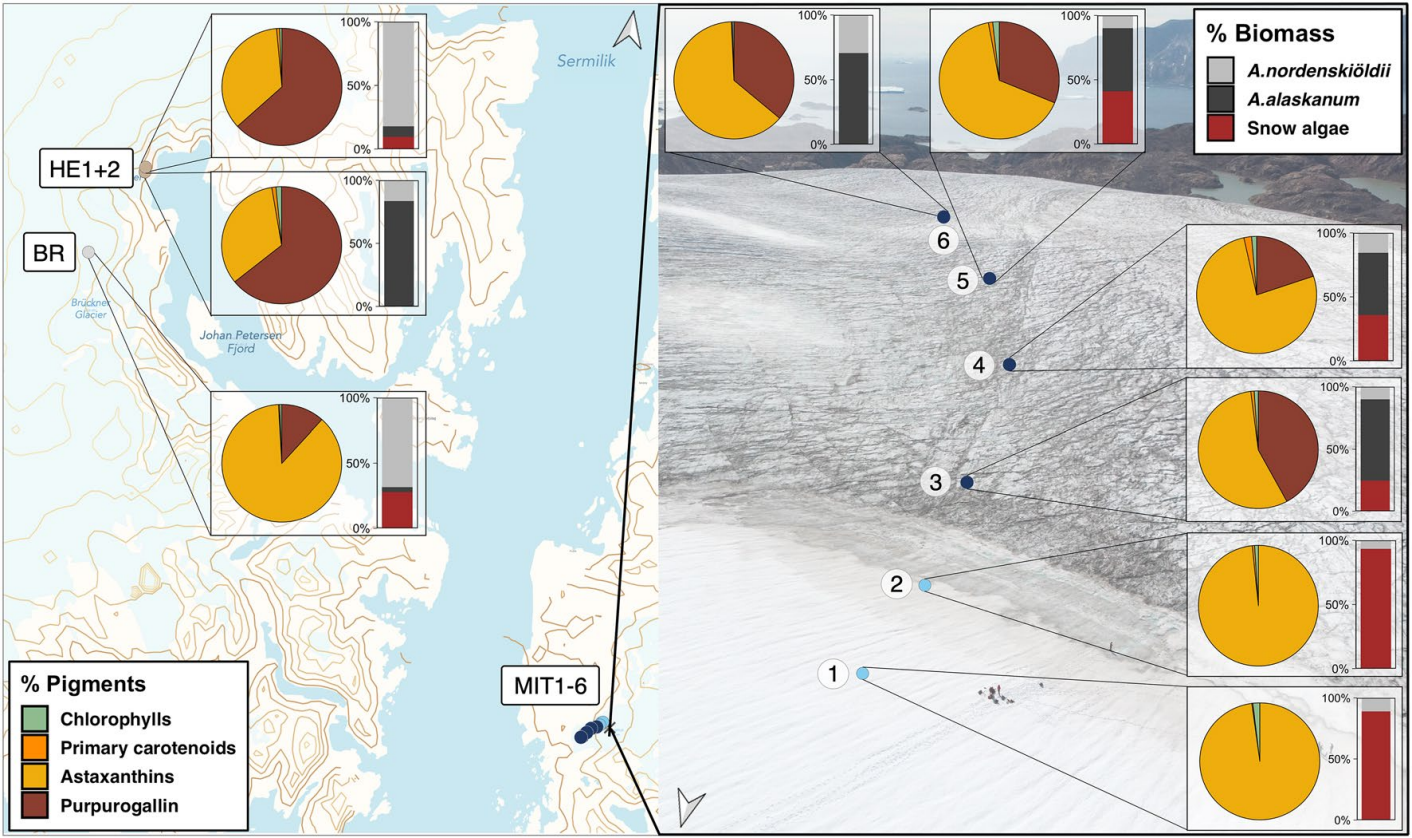
Pigment composition and relative biomass contribution of algal groups at different locations on glaciers on Greenland. Pie charts show the contribution of various pigment groups to the total pigment pool in % as different colours. The pigments were grouped as: chlorophylls (chl *a* and *b* and pheophytin *a*), primary carotenoids (PPC; violaxanthin, neoxanthin, lutein, zeaxanthin), astaxanthins (*trans*-astaxanthin, *trans*-and *cis*-astaxanthin esters) and purpurogallin (comprising different derivative forms of purpurogallin). The individual contributions of *A. nordenskiöldii, A. alaskanum* and snow algae to total community biomass in % are shown as different colours in the bar charts. Data represent averages of three samples collected at each site. For % values see Supplementary Information Table S5. Map in (**a**) was created using QGIS v.3.16 (http://www.qgis.org)^78^ and background in (**b**) shows a drone photo of the sampling transect on Mittivakkat.

The pigment compositions of the algal community are presented as % of the pigment pool and as molar ratios to chlorophyll *a* (chl *a*) (Fig. 4 and Table 1; see Supplementary Table S5 for % values and Table S6 for mass concentrations, in mg L^−1^). LC-HRMS/MS-screening confirmed the presence of both purpurogallin-carboxylic-acid and purpurogallin-carboxylic-acid-glycopyranoside (Supplementary Methods), with the latter present in higher quantities^18^. Due to their similar λ_max,_ we summarise both as purpurogallin hereafter. The community pigment pool was primarily dominated by purpurogallin as well as astaxanthin esters. Their concentrations were significantly positively correlated with the biomass of their respective algal hosts (R^2^ = 0.5, p < 0.001, n = 22; R^2^ = 0.53, p < 0.01, n = 18, respectively). The chl *a* concentrations were also significantly positively correlated with biomass (R^2^ = 0.56, p < 0.001, n = 25; respectively). Other pigments were only found in low concentrations.

**Table 1:** Overview of average chlorophyll *a* (chl *a*) concentrations (mM) and molar ratios with other pigments per site: *Pheo* = pheophytin *a*, *Neo* = neoxanthin, *Vio* = violaxanthin, *Lut* = lutein, *Zea* = zeaxanthin, *PPG* = purpurogallin, *Ast* = astaxanthin, *Ast-ester* = astaxanthin fatty acid derivatives (mono and di esters) and *GA:SA* = biomass ratio of glacier ice and snow algae.

Site	Chlorophylls				Primary carotenoids								Phenols		Secondary carotenoids										Biomass
	Chl *a*		Pheo:Chl *a*		Neo:Chl *a*		Zea:Chl *a*		Vio:Chl *a*		Lut:Chl *a*		PPG:Chl *a*		*Trans*-Ast:Chl *a*		*Trans*-Ast-mono-ester:Chl *a*		*Trans*-Ast-di-ester:Chl *a*		*Cis*-Ast-mono-ester:Chl *a*		*Cis*-Ast-di-ester:Chl *a*		GA:SA
	Mean	Range	Mean	Range	Mean	Range	Mean	Range	Mean	Range	Mean	Range	Mean	Range	Mean	Range	Mean	Range	Mean	Range	Mean	Range	Mean	Range	mean
BR	1.5E−04 ± 6.8E−05	9.7E−05–2.2E−04	0.49 ± 0.85	0–1.47	0.31 ± 0.24	0.06–0.53	0.06 ± 0.11	0–0.19	0.16 ± 0.12	0.04–0.28	0.00	0–0	19.45 ± 9.67	9.03–28.11	1.08 ± 0.22	0.93–1.33	22.62 ± 10.23	25.67–30.97	16.68 ± 5.04	11.31–21.31	89.26 ± 12.72	77.98–103.05	24.94 ± 8.30	17.48–33.87	2.73
HE1	4.4E−06 ± 3.7E−06	1.7E−06–7.1E−06	0.00	0–0	0.37	0.52–0.22	0.08	0.04–0.12	0.56 ± 0.27	0.74–0.37	0.00	0–0	263.51 ± 372.66	0–527.02	0.21	0.20–0.22	17.50	12.19–22.81	5.76	5.43–6.09	11.36	5.15–17.55	1.04	0.22–1.86	16.57
HE2	4.9E−06 ± 5.6E−06	2.3E−07–1.1E−05	0.00	0–0	0.12 ± 0.06	0.14–0.05	0.01 ± 0.01	0–0.02	0.20 ± 0.05	0.15–0.25	0.12 ± 0.19	0–0.012	155.37 ± 212.13	25.55–400.17	0.03 ± 0.05	0–0.08	5.90 ± 5.28	1.83–11.87	12.14 ± 10.05	4.68–23.56	8.91 ± 12.38	0.17–23.08	5.68 ± 5.90	0.73–12.2	Only GA
MIT1	4.4E−06 ± 3.0E−06	1.5E−06–7.5E−06	0.00	0–0	0.00 ± 0.00	0–0	0.00	0–0	0.00	0–0	0.19 ± 0.14	0.06–0.34	0.00	0–0	0.03 ± 0.03	0–0.05	4.09 ± 1.27	2.91–5.42	9.10 ± 2.34	7.72–11.80	10.52 ± 8.29	0.98–16.01	28.05 ± 11.85	16.62–40.27	0.49
MIT2	3.8E−05 ± 3.2E−05	1.7E−05–7.4E−05	1.19 ± 0.50	0.72–1.71	0.11 ± 0.07	0.05–0.19	0.04 ± 0.07	0–0.13	0.12 ± 0.14	0–0.27	0.20 ± 0.26	0–0.49	0.00	0–0	0.05 ± 0.02	0.03–0.07	8.27 ± 4.22	3.73–12.06	11.84 ± 5.15	8.31–17.75	33.31 ± 13.33	19.38–45.96	62.89 ± 22.51	57.55–88.73	0.07
MIT3	5.7E−05 ± 4.4E−05	1.6E−05–1.0E−04	0.54 ± 0.36	0.15–0.86	0.10 ± 0.03	0.07–0.13	0.07 ± 0.06	0–0.11	0.13 ± 0.06	0.07–0.19	0.65 ± 0.18	0.49–0.61	63.82 ± 48.09	27.67–118.40	0.32 ± 0.28	0–0.54	25.04 ± 4.72	20.55–29.96	4.27 ± 0.83	3.47–5.13	29.49 ± 2.93	26.56–32.42	0.41 ± 0.39	0.05–0.83	13.14
MIT4	5.3E−05 ± 1.4E−05	3.7E−05–6.3E−05	0.42 ± 0.30	0.22–0.76	0.44 ± 0.20	0.21–0.57	0.15 ± 0.26	0–0.45	0.58 ± 0.20	0.41–0.80	0.98 ± 0.40	0.65–1.42	24.85 ± 30.42	4.02–59.76	1.37 ± 0.62	0.98–2.08	31.51 ± 7.34	23.12–36.71	5.97 ± 2.24	4.16–8.45	37.17 ± 10.23	25.36–43.43	0.73 ± 0.47	0.19–1.02	1.68
MIT5	9.1E−05 ± 2.7E−05	6.0E−05–1.1E−04	0.27 ± 0.08	0.21–0.36	0.15 ± 0.21	0–0.39	0.14 ± 0.13	0–0.25	0.20 ± 0.19	0.03–0.4	0.54 ± 0.16	0.44–0.72	26.88 ± 27.54	4.3–57.57	0.96 ± 0.40	0.60–1.38	23.80 ± 10.47	12.12–32.34	3.83 ± 0.37	3.49–4.22	30.19 ± 6.72	23.45–36.88	0.43 ± 0.30	0.18–0.76	1.75
MIT6	9.5E−06		0.92		0.04		0.08		0.18		0.32		108.15		0.07		56.61		85.73		45.27		0.77		Only GA

Data are presented as averages per site followed by the standard deviation (n = 3).

See Supplementary Material for concentrations in mg L^−1^ (Table S6).

Where the glacial ice algae-derived purpurogallin was detected, its contribution to the pigment pool varied on average between 12% at Bruckner Glacier to 64% at Heim Glacier (HE2), while no purpurogallin was present on the snow sites on Mittivakkat (MIT1 + 2) (Fig. 4, Supplementary Table S5). The purpurogallin:chl *a* ratio was on average 20, 56 and 209 at Bruckner Glacier, the bare ice sites on Mittivakkat (average of MIT3-6) and Heim Glacier (average of HE1 and HE2), respectively. Cellular purpurogallin contents varied from 0.01 to 0.02 ng cell^−1^ within a 95% CI with an average concentration of 0.023 ng cell^−1^ and did not significantly differ between sites. The log-transformed purpurogallin content per cell showed a positive correlation with biovolume, though not significantly (R^2^ = 0.49, p = 0.074; Supplementary Figure S6).

Astaxanthin and its esters were present in all samples and dominated the overall pigment pool between 33 to 98%. This was particularly prominent on the snow sites BR and MIT1 and MIT2, where they constituted 87 and 97–98%, respectively, of the total pigment pool. Astaxanthin esters still made up 33 to 77% of the total pigment pool on the bare ice sites on Mittivakkat (MIT3-6) and Heim Glacier, despite comparably low snow algal biomass. *Cis*-astaxanthin monoesters were the dominant astaxanthin esters on the snow sites and were less abundant in bare ice snow algal communities, resulting in a lower *trans* to *cis*-astaxanthin ester ratio at those three sites. We also found traces of a phenolic compound in the hydrophilic extracts of the snow algal-dominated snow sites on Mittivakkat (Supplementary Fig. S1 and S3), absorbing at 280 nm, which was identified as a gallic acid glycoside^46^.

### Chromophoric dissolved organic matter

**Figure 5:**
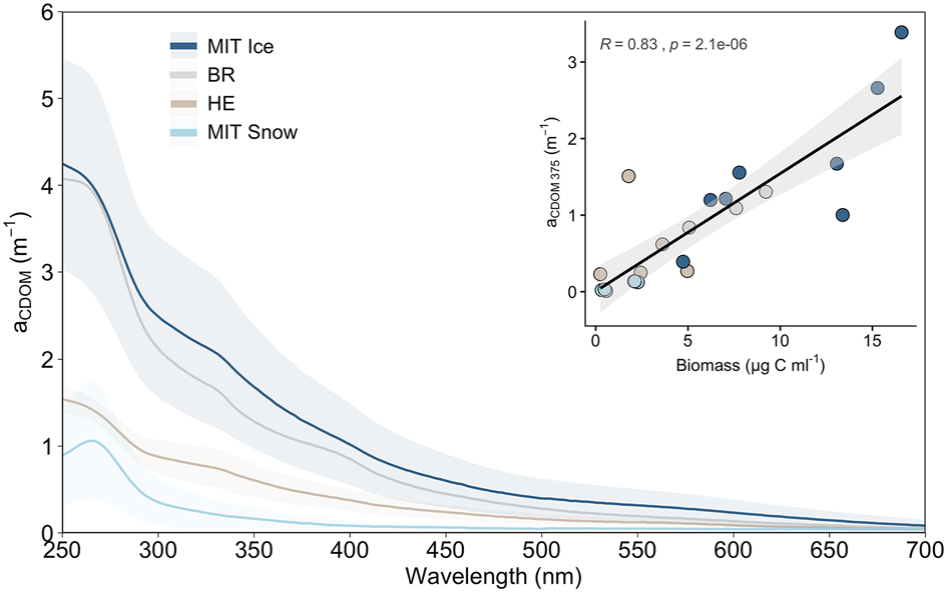
Chromophoric dissolved organic matter (CDOM) spectral absorption among regions. Insert shows the correlation of a_CDOM 375_ with total algal biomass. Grey area indicates the 95% CI and R the Spearman correlation coefficient.

*a*_CDOM __375_ absorption is used as a proxy for CDOM concentration and ranged between 0.08 m^−1^ on the snow (average of MIT1 + 2) to 1.38 m^−1^ on the bare ice sites on Mittivakkat (average of MIT3-6; Fig. 5, concentrations shown in Supplementary Table S7). We found a significant positive correlation between *a*_CDOM __375_ and algal biomass (R^2^ = 0.83, n = 23, p < 0.01; insert Fig. 5). Absorption spectra displayed a shoulder at around 335 nm in all regions, except at the snow sites at Mittivakkat and we identified purpurogallin in the dissolved fraction of glacier ice by LC-HRMS/MS screening (Supplementary Methods). Using the average DOC concentration of 1 mg L^−1^ for the bare ice sampling sites on Mittivakkat gives an average mass-specific absorption coefficient for DOC_365_ of 1.46 m^2^ g^−1^ (range 0.88–2.66 m^2^ g^−1^, Supplementary Fig. S7). This CDOM absorption coefficient can be incorporated in a radiative transfer model but physical ice parameters and CDOM vertical distribution within the ice surface (homogeneous or up-concentrated only in the upper mm) must be better constrained to be able to assess the impact of CDOM on albedo reduction.

### Algal absorption properties and impact on radiative forcing

**Figure 6:**
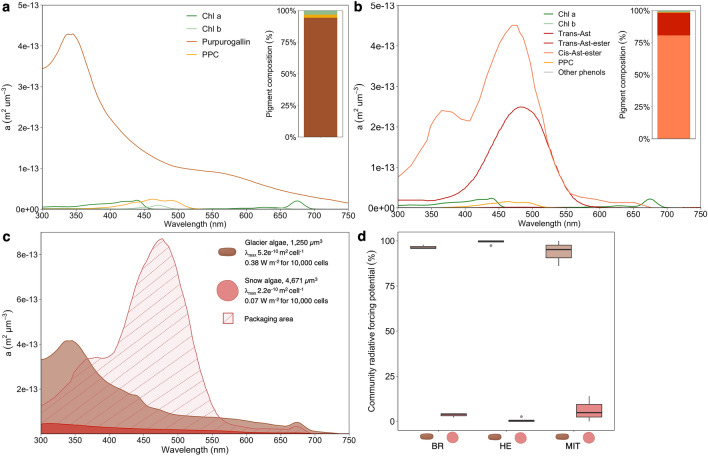
Reconstructed and measured biovolume specific absorption coefficients of glacier ice and snow algae and estimated radiative forcing. (**a**) Reconstructed spectral absorption of glacier ice algae for each pigment group of the average pigment composition (barplot insert). (**b**) Reconstructed spectral absorption of snow algae for each pigment group of the average pigment composition (barplot insert). (**c**) Reconstructed absorption of glacier ice algae (brown colour) and snow algae (red area without filling) with additionally measured “packaged” cellular absorption of snow algal cells (red colour with the filled area). For snow algae, the difference between the reconstructed approach and the “packaged” cellular absorption is marked as the striped area. Instantaneous radiative forcing per algal group estimated for the incoming irradiance of 91,763 W m^−2^ (**b**) Percentage contributions of glacier ice and snow algae to instantaneous radiative forcing of the algal community for the community composition scenarios at Bruckner Glacier, Heim Glacier and Mittivakkat. Cell shapes were created with BioRender.com.

To study the role of individual pigment groups in algal absorption, we reconstructed pigment-derived absorption coefficients (Fig. 6a,b) using the absorption spectra of isolated pigments (Supplementary Fig. S3) and their intracellular concentrations. The reconstructed glacier ice algal absorption was dominated by purpurogallin and peaked at 338 nm (Fig. 6a). In contrast, the snow algal absorption spectrum was largely dominated by astaxanthin-esters in *trans* (absorbing at 470 nm) and *cis* conformations (absorbing at 468 and 378 nm; Fig. 6b). The additionally measured “packaged” cellular absorption spectra of snow algae (Fig. 6c) showed a strong flattening effect, hiding the astaxanthin ester absorption peaks. Snow algal cellular absorption including packaging (λ_max_ 2.2 × 10^−10^ m^2 ^cell^−1^ and 4.73 × 10^−14^ m^2^ µm^−3^) was lower than the expected pigment-derived absorption (λ_max_ 4.07 × 10^−9^ m^2 ^cell^−1^ and 8.71 × 10^−13^ m^2^ µm^−3^), resulting in a 95% lower total absorbed irradiance. Using the literature spectra for glacier ice algae including packaging (λ_max_ 4.25 × 10^−14^m^2^ µm^−3^ and 9.76 × 10^−10^ m^2^ cell^−1^, see “Method” section), we compared glacier ice algal absorption with snow algal absorption including packaging. Integrated over the incoming solar irradiance, glacier ice algal cellular absorption exceeds snow algal absorption by a factor of 5 (0.38 vs. 0.07 W m^−2^ for 10,000 cells, respectively). Their respective contribution to the total radiative forcing of the algal community was estimated depending on their biomass for each site (Fig. 6d), showing that snow algae contributed on average to 4 ± 4.2% (range 0-14%).

## Discussion

### Spatial patterns in algal community composition are linked to photophysiological performances

We observed widespread algal blooms on bare ice and snow surfaces of three different glaciers in SE-Greenland. Traditionally, glacier ice and snow algae are mostly studied separately because they dominate their respective habitats. However, our data show that community mixing occurs upon snowmelt, so that snow algae still contributed to 44% of the total algal biomass on the bare ice. This is in line with the study by Lutz et al.^32^, who showed that snow algal biomass even exceeded glacier ice algal biomass locally on the same glacier. Bare ice snow algal abundance varied across the altitudinal gradient on Mittivakkat, likely reflecting local snow dynamics (including snow depth and snowmelt rates) controlling the presence of snow algae on snow^47^ and ultimately also on bare ice upon snow melt. Likewise, glacier ice algae can also be found in surprisingly large numbers on snow^32^. On Bruckner Glacier, they contributed up to 80% of the total community biomass. Since glacier ice algae are not motile, atmospheric deposition of cells could have transported them onto the snow surface or the local conditions of the snow cover being only ~ 10 cm thick and having a frozen crust may have provided favourable growth conditions for glacier ice algae so that they became dominant over time.

Overall, the intra-site variability in total algal abundance was higher (MIT3-5, on average 36%) than the inter-site variability of the same glacier and between glaciers, despite the different growth conditions (e.g., marine- vs. land-terminating, slope exposure, altitude, ablation rates). Therefore, local mobilisation and redeposition processes associated with a dynamic weathering crust and supraglacial meltwater flows^48–50^ can impact algal cell accumulation already on a metre scale and drive local ecological niche formation. Note that due to the restricted accessibility of Heim and Bruckner Glaciers, the number of samples that could be collected there was limited and lower than on Mittivakkat, which could represent a potential caveat for the inter-site comparisons. Despite the high within-site variability, we could still observe large changes in algal abundance along the snow to bare ice transect on Mittivakkat. There, total algal cell numbers increased with decreasing altitude, reaching the highest average concentrations at 343 m elevation (MIT5). Meltwater drainage downslope^51^ has been shown to drive algal accumulation downstream on the GrIS^52–54^, hence likely accounting at least partly for higher cell numbers at sites of the lowest altitude in our study. An extended growth period due to an earlier snow retreat at sites of lower altitudes could additionally explain the higher algal numbers at the lower altitudes, considering the estimated ~ 13 days delay of snow retreat for an altitude difference of 113 m (MIT3-6)^55^.

The spatial variability in algal diversity implies variable community photobiology across sampling sites. Accordingly, we found substantial differences in the photophysiology between snow and bare ice communities originating from the same glacier. The significantly lower F_v_/F_m_, rETR_max_ and α of the snow community (MIT1 + 2) suggest lower photosynthetic performances including a lower efficiency in utilising light compared to the bare ice community (MIT3 + 4). This may be attributed, at least partly, to the different physiological states of the algal groups: typical snow algae such as *Chloromonas nivalis* dominate the snow community in its dormant cyst stage during the melt season^23^ of which the F_v_/F_m_ was shown to decrease throughout the summer^56^, whereas glacier ice algae have no dormant stage and can actively divide throughout the season^57^. Thus, during the late summer melt season, the snow communities may not need to invest heavily into their photosynthetic capacity, compared to glacier ice algae remaining in their vegetative stage. In addition, solar exposure at both landscape and micro-niche scales can also impact algal photophysiology. Since Mittivakkat faces more south than Heim Glacier (Fig. 1a), the increased solar exposure may explain the significantly higher rETR_max_ and F_v_/F_m_ of the bare ice sites on Mittivakkat compared to Heim Glacier.

### Purpurogallin and astaxanthin esters dominate algal pigment signatures during a summer melt season

Blooms of glacier ice and snow algae darken the GrIS due to their intracellular accumulation of secondary pigments^7,10,15,32^. On glaciers, purpurogallin is the pigment unique to glacier ice algae^17,18^, astaxanthin and its fatty acid ester derivatives are only produced by snow algae^20,58^ and are particularly abundant in their mature cysts^24,25^. Since studies on mixed communities did not analyse full hydrophilic (purpurogallin dominated) and lipophilic pigment pools^32^, our understanding of algal community pigmentation on glaciers where snow and glacier ice algae co-occur is limited.

We show that purpurogallin and astaxanthin esters dominated algal community pigment pools (on average 4 and 95% on snow as well as 43 and 55% on bare ice, respectively) and each pigment group served as a biomass indicator for its respective algal host. Due to the high intracellular concentrations of astaxanthin esters, snow algae had a proportionally large contribution to community pigment pools, despite their smaller contribution to biomass compared to glacier ice algae. The total astaxanthin ester:chl *a* mass ratios of our snow algal samples were on average higher (56) compared to previously documented ratios, e.g., 34 for cells from Svalbard in Müller et al.^22^ and 50 in Lutz et al.^32^ for cells from Mittivakkat. This could be due to seasonal pigment variability or composition of the snow algal community, where other species of snow algae that are less pigmented dilute the astaxanthin ester pool relative to chl *a*^59^. Note that we can’t exclude a potential impact on the obtained pigment concentrations by the melting of the ice and snow samples in the dark prior sample preservation. In addition, varying environmental conditions may account for some of the observed differences in pigment concentrations compared to other studies.

While the pigment regulation mechanisms in snow algae have been studied by field observations and laboratory experiments in numerous studies^21,46,60,61^, empirical data for glacier ice algae are scarce^15,19^, restricting our understanding of their pigment regulation and consequently their ability to darken the ice surface. Several factors should be considered for assessing the active intracellular up-or down-concentration of the purpurogallin pool, of which we discuss the first three in more detail below: (i) duration of growth period/snow retreat^19^; (ii) photo-acclimation aiming to ensure efficient light capture while simultaneously avoiding damaging-effects of excessive solar radiation (^62,63^ for other microalgae); (iii) cell size and timing since cell division^41,64^; (iv) antimicrobial activity (as shown for benzotropolone derivatives^65^); and (v) adaptation to low temperature by generating heat for ice and snow melt^15,26^. We expected an increasing purpurogallin:chl *a* ratio with decreasing altitude, assuming that altitude is a proxy of time since snow retreat with a consequent higher exposure to irradiance at lower latitude. However, the highest purpurogallin:chl *a* ratio was found on Heim Glacier located at only 286 m of altitude, while it was ~ 11 × lower on Bruckner Glacier with the highest altitude of 942 m. Overall, the average cellular purpurogallin concentrations (0.03 mM cell^−1^, 0.023 ng cell^−1^) were comparable to the bulk phenol measurements by Williamson et al.^15^ reporting an average of 0.041 ng cell^−1^, underlining that purpurogallin is the major phenolic compound^18^ and demonstrating similar pigment content between West and East Greenland glacier ice algae. Moreover, the cellular and biovolume normalised purpurogallin content did not show any correlation with photophysiological performances across glaciers, suggesting that there is no direct link between the two under in situ conditions. This matches the observation of Williamson et al.^15^ that glacier ice algae are photo-inhibited during summer and, hence, a downregulation of the purpurogallin stock would in fact increase their risk of photo-damage. Newly divided, smaller cells may have generally a lower photoprotective pigment content compared to older, larger cells as shown for other Zygnematophycean algae^41^ or phytoplankton^64^, which could also apply to glacier ice algae as shown by the increasing trend of cellular purpurogallin concentration and biovolume (Supplementary Information, Fig S6). Apart from active pigment regulation mechanisms in the algae, their cellular purpurogallin content may also be indirectly altered through infections of parasitic fungi, which are widespread on the GrIS^66,67^.

Cell leakage of pigments could also impact CDOM signatures^27^ and play a role in ice and snow optical properties and UV-light exposure of algal cells, as demonstrated for sea-ice habitats^30,68^. We found that CDOM in our study was significantly linked to algal biomass, suggesting a direct or indirect link between them by either releasing light-absorbing metabolites, cell leakage or bacterial degradation of particulate organic matter surrounding the algae. This is in line with studies reporting the close link between algal community assemblages and DOC on Sweden and Svalbard glaciers^69^ and bacterial communities and the quality and quantity of DOM from Antarctica and Greenland^70^ from various glacial habitats. In our study, the spectral characteristics of CDOM demonstrated potential purpurogallin signatures in the environment (Fig. 5), which was indicated by an absorption shoulder at λ_338_ only present at glacier ice algal-dominated sites. High-resolution LC-HRMS/MS screening further verified the presence of this pigment, as purpurogallin carboxylic acid-6-O-b-D-glucopyranoside, in the dissolved fraction (Supplementary Methods). Hence, purpurogallin could be an important constituent of the DOM pool on glacier surfaces.

### A pigment-derived perspective on algal absorption and impact on surface darkening

We studied the snow and glacier ice algal pigment signatures in order to decipher their role in energy absorption during a late summer melt season. We hypothesised that the contrasting secondary pigments:chl *a* ratios of glacier ice and snow algae outlined above and the different absorption properties of their individual pigments would result in higher energy absorption of snow compared to glacier ice algae. Although astaxanthin esters in *trans* and *cis* conformations absorb stronger than purpurogallin at mid-visible wavelengths where incoming irradiance peaks (Supplementary Fig. S3), we showed that glacier ice algae are overall more effective absorbers than snow algae. Their average per cell energy capture is ~ 5 × higher than that of snow algae. This is at least partly due to a “pigment packaging effect” that is particularly pronounced in snow algae (Fig. 6c), which is in line with the study by Chevrollier et al.^16^ from south-west Greenland. Depending on cell size, internal structure and presence of pigment-protein complexes, the absorption of “packaged” pigments (i.e., inside the cell) can be lower compared to the potential absorption of the same amount of pigments in solution^71–73^. While all algal cells store the pigments intracellularly and, hence, “package” their pigments, the different strength of this effect in both algal groups is likely related to the generally stronger packaging in larger than smaller cells^72^ and spherical (snow algae) compared to cylindrical (glacier ice algae) cells^74^. Thus, a combination of these factors likely explains the difference in magnitude and shape (flat versus spiky spectrum) between measured “packaged” and theoretically reconstructed algal absorption spectra^75^. For snow algae, variability in cellular light absorption can be expected between species, individual cells^76^ and physiological stages of the same species (green pigmented vegetative cells or red pigmented cysts)^12^. The absorption spectra obtained in our study are therefore likely most representative of red-pigmented snow algal cysts (*Chlamydomanas* cf*. nivalis*).

Snow algae have been shown to contribute significantly to albedo decline on snow^10–12^ and even though their occurrence on bare ice has been reported^32,54^, their impact on bare ice darkening after snow retreat has not yet been studied. We demonstrate that within a mixed bare ice community, glacier ice algae remain the main energy absorbers and snow algae contribute only minimally (on average 4%) to the total radiative forcing driven by algae (Fig. 6d). Their contribution to the total algal community radiative forcing may vary depending on their biomass (Bruckner Glacier, Heim Glacier and Mittivakkat showed on average contributions of 6, 1 and 4%, respectively, range 0–14%), pointing to glacier-wide differences in snow algal contribution to algal energy capture and, hence, albedo reduction. Nevertheless, locally snow algae can be found at higher concentrations on bare ice^32^, which is supported by observations of bare ice areas on Mittivakkat, which appeared red and showed absorption spectra that are typical for snow algae (Supplementary Fig. S9). The regional variability of snow algal biomass on bare ice is likely tightly linked to their earlier bloom development on snow, which can be characterised by considerable spatial and temporal heterogeneity. For example, they have been reported on the GrIS close to Tasiilaq (this study), Ilulissat (personal observation), Narsarsuaq^77^ or Qaanaaq^47^, whereas they were reported absent close to Kangerlussuaq^14,19^. The radiative forcing by snow algal populations is expected to vary depending on the snow algal community composition and/or pigmentation^12^. Over a season, the cumulative impact on meltwater generation of each alga will vary depending on meteorological factors such as the longevity of the snowpack, local temperature hydrology and irradiance conditions, and nutrient availability for each habitat.

## Conclusions

By sampling three different Greenlandic glaciers, we have found a variable algal community composition with red snow algal cysts being largely present on bare ice habitats. This snow and bare ice community species mixing has so far been rarely considered and has implications for bare ice algal biomass accumulation and bare ice albedo. The snow and glacier ice algal relative abundances manifest in distinct pigment signatures, which are dominated by glacier ice algae-derived purpurogallin and snow algae-derived astaxanthin esters. These photoprotective pigments are key to algal photoprotection and, thus, energy absorption. Algal pigment pools do not mirror respective snow and glacier ice algal contribution to radiative forcing due to a pigment packaging effect. This effect is particularly pronounced in snow algae and lowers their cellular absorption by 95% compared to the expected pigment-derived absorption. We reveal that glacier ice algae are overall ~ 5 × more effective energy absorbers compared to snow algae. Of the total radiative forcing driven by algae on the bare ice, the contribution by snow algae remains on average small (4%) but may locally be higher (up to 14%). At the glacier scale, this indicates a spatio-temporal effect of biological albedo reduction synchronous with the retreat of a seasonal snowpack: snow algae are the main albedo reducers on wet snowpacks and once melted, glacier ice algae become the dominant albedo reducer even while snow algal cells remain on the bare ice. Our results highlight the role of community mixing during the snow melting season for algal biomass accumulation and bare ice community energy absorption. Future research using in situ albedo measurements and pre-and post-melt algal biomass will help to elucidate the role of community composition for surface albedo reduction in more detail.

## Supplementary Information

Supplementary Information.

## Data Availability

The data set of this study can be found in the Supplementary Information.
