# Supplementary material for: Pigment signatures of algal communities and their implications for glacier surface darkening
Source: Sci Rep. 2022 Oct 21;12:17643. doi: 10.1038/s41598-022-22271-4 (PMC9587043; doi:10.1038/s41598-022-22271-4)
Supplement: Supplementary file 1 — Supplementary Information. [file 41598_2022_22271_MOESM1_ESM.pdf]

Supplementary Information

Pigment signatures of algal communities and their implications for glacier surface darkening

Laura Halbach*, Lou-Anne Chevrollier, Eva L. Doting, Joseph M. Cook, Marie B. Jensen, Liane G. Benning, James A. Bradley, Martin Hansen, Lars C. Lund-Hansen, Stiig Markager, Brian K. Sorrell, Martyn Tranter, Christopher B. Trivedi, Matthias Winkel, and Alexandre M. Anesio

Method details

Site information

Table S1. Sample site information. MIT: Mittivakkat glacier, BR: Bruckner Glacier, HE1+2: Heim Glacier.

Site	Date	Lat	Long	Altitude	Glacier	Substrate	Colouration
MIT1	190724	N 65°41'39"	W 37°50'02"	451 m	Mittivakkat	Snow	White/red
MIT2	190724	N 65°41'40"	W 37°50'05"	445 m	Mittivakkat	Snow	Reddish
MIT3	190724	N 65°41'38"	W 37°50'25"	415 m	Mittivakkat	Ice	Dark
MIT4	190725	N 65.69266	W 37.85115	370 m	Mittivakkat	Ice	Dark
MIT5	190727	N 65°41'26"	W 37°51'26"	343 m	Mittivakkat	Ice	Dark
MIT6	190721	N 65°41'21"	W 37°51'50"	274 m	Mittivakkat	Ice	Dark
BR	190726	N 65°57'1"	W 38°31'8"	942 m	Above Bruckner	Snow (refrozen)	White
HE1	190726	N 65°59'35"	W 38°26'50"	286 m	Heim	Ice	Dark
HE2	190726	N 65°59'35"	W 38°26'50"	286 m	Heim	Ice	Medium dark

Environmental data retrieved from PROMICE stations

Table S2. Environmental parameter retrieved from PROMICE weather stations "MIT" located on Mittivakkat glacier at 440 m altitude and "TAS_A" close to Bruckner Glacier at 890 m altitude. Values present averages for the sampling period July 21-27 for the "MIT" station and for "TAS_A" for July 26 2019. The UV-A and UV-B radiation on Mittivakkat were measured by us using a radiometer (SolarLight, PMA2100) during noon on July 24.

PROMICE station	Air temperature (°C)	Daily incoming shortwave radiation (W m^{-2})	Ablation rate (mm day^{-1})	Broadband albedo	UV-A and UV-B radiation* (W m^{-2})
MIT	4.0 (range: 2.5-6.4)	227 (range: 63-346)	18.9	0.25 (range: 0.23-0.28)	33 and 2.8
TAS_A	3.1 (range:1.3-4.6)	334 (range: 212-374)	32.2		

PAM fluorometry

For Phyto-PAM measurements, the Phytowin software Version 1.45 was used. Measurements were performed against filtered (0.2 µm PTFE) blank subsamples of each sample. After optimising the sensitivity (gain) of the instrument for our sample type (using a sample with representable algal concentration), photosynthetic quantum yields were measured. Minimum (Fo) and maximum fluorescence (Fm) yields were measured by applying a weak non-actinic light (i.e., not enough to stimulate photosynthesis) followed by a short saturation light pulse of 4000 µmol m^{-2} s^{-1}, respectively. Using the fluorescence yield after dark adaptation (30 min), the maximum quantum efficiency (Fv/Fm) was calculated (equation 1). The quantum yield (equation 2) can be used to calculate the relative electron transport rate (rETR) (equation 3), which is a proxy for the rate of electrons channelled through the photosynthetic chain of photosystem 2 (PS II, assuming an equal electron transport rate of PSI and PSII) (Beer et al. 2001). Therefore, we used a series of eight short light pulses to assess the algae's photosynthetic

activity in light exposed cells (0, 8, 16, 32, 64, 164, 364, 564, 764, 1964, 1364, 1764 and 2064 µmol m^{-2} s^{-1}). Different photosynthesis vs. irradiance (P/I) curves (expressed as rETR vs. Photosynthetic active radiation (PAR)) were evaluated for our data and the model by Jassby and Platt (1976) was subsequently fitted onto the rapid light curves (RLC) using the phytotools package (Greg and Malkin, 2015) in R (v. 3.6.0.). From the RLC curves other photosynthetic parameters such as the light utilization coefficient (i.e., slope of RLC curve where P is limited by light (alpha)), the light saturation coefficient (Ek) and the maximum relative electron transport rate (rETRmax) were calculated:

$$Fv/Fm = (Fm - Fo)/Fm \quad (1)$$

$$Y(PSII) = (F^{\wedge\prime}m - F)/F'm \quad (2)$$

$$rETR = Y(PSII) \times incident\ excitation \times 0.5 \quad (3)$$

Pigment extractions

To characterise and quantify major lipophilic and hydrophilic pigments from algal cells concentrated on filters, a two-phase extraction was performed using a protocol adapted from Aigner et al., (2013b) and Holzinger et al. (2018) and while working under reduced light and on ice whenever possible. Filters were placed into 2 ml bead-beating tubes (Lysing Matrix E, MP Biomedicals, France) and freeze-dried in the dark for 24 hrs. Thereafter, 800 µl of methyl tertiary-butyl ether (MTBE) was added and cells were disrupted on a bead-rupter (60 s, 6 m s^{-1}; Omni International, Kennesaw, GA), cryo-cooled with liquid nitrogen down to 5 °C. The filters were subsequently shock frozen in liquid nitrogen for 10 seconds to further improve cell lysis. Bead-beating and shock freezing was repeated 3 more times (in total 4 times) followed by an immersion of the tubes in an ultrasonic bath for 15 minutes at 0-5 °C to initiate additional cell lysing. After centrifugation (10.000 *g*, 4 °C, 5 min), the supernatant was collected and 400 µl fresh MTBE was added again in addition to 500 µl 20 % methanol (MeOH). The tubes were vortexed and frozen overnight at -20 °C. Thereafter, samples were defrosted, homogenized on the cooled bead-beater (20 s, 6 m s^{-1}) and again centrifuged (8 min) to allow phase separation. The MTBE and MeOH phases were collected into separate Eppendorf tubes (one of them already contained the first MTBE extract). As a modification from the protocol described by the two studies mentioned above, we then added again 20 % MeOH (800 µl), followed by 20 s of beat-beating and centrifugation (10.000 *g*, 4 °C, 5 min). This means we used in total 2 extraction steps per phase to improve the extraction efficiency, of which the importance was visually confirmed by yielding coloured extracts. The MTBE and MeOH phases were subsequently freeze-dried for approx. 4 hours, respectively, and the former phase was reconstituted in 250 µl 100% acetone, while the latter phase was reconstituted in 50% MeOH.

Pigment analysis

Lipophilic pigments were analysed on a high-pressure liquid chromatography (HPLC; Diode array detector, Shimadzu) following (Wright, 1991), using a C18 column (Supelco, 250x4.6 mm, 5 µm). Pigments were quantified using a series of standards and at the wavelengths matching their absorption maxima: Carotenoids at 440 nm (standards for Neoxanthin, Violaxanthin, Luthein obtained from DHI, Denmark), Chlorophylls at 662 nm (standards for Chlorophyll *b*, Chlorophyll *a*, Phaeophytin obtained from DHI, Denmark) and *all-trans-*

astaxanthins (obtained from Carotenature, Switzerland) and its esters at 470 nm. Astaxanthin ester concentrations were estimated by correcting conservatively for the mass difference between *all-trans*-astaxanthin and its respective mono-and diesters bound to oleic acid, which was reported in Bidigare et al. (1993) as the most abundant fatty acid in red C. nivalis cells. This step was necessary to avoid the overestimation of their mass specific concentration due to their much higher mass compared to their non-esterified counterparts. Note that the fatty-acid esters do not interfere with the chromophore's absorption, and thus, absorb at the same wavelengths as astaxanthin itself, but can be differentiated using the retention time differences reported in Remias and Luetz (2007).

Table S3. Average, minimum and maximum retention times for the major quantified pigment classes following Wright (1991). Neo= Neoxanthin, Viola= Violaxanthin, Lut= Lutein, Zea=Zeaxanthin, Chl= Chlorophyll, Asta=Astaxanthin and Pheo=Pheophytin a.

	Neo	Viola	Anthera	Trans-Asta	Lut	Zea	Chl b	Chl *a*	Trans-Asta-esters	*Cis*-Asta esters	Pheo
**RT average**	12.5	13.43	15.78	17.05	17.23	17.84	23.71	24.94	28.59	27.96	26.7
RT min	12.1	13	15.33	16.03	16.6	17.14	23	24.72	26.507	23.61	26.4
RT max	13.6	14.04	17.12	18.16	18.1	19	24.82	25.55	31.93	31.32	27.3

To estimate the contents of the water-soluble pigment, purpurogallin, the absorption of the hydrophilic extract fraction was measured on a microvolume spectrophotometer (220-750 nm, 1 nm intervals, Nanodrop 200/2000c, Thermofisher, Germany). To do this, extracts were centrifuged (10.000 *g*, 10 min) and 1.5 µl aliquots were measured on the pedestal of the instrument by performing a minimum of 3 replicate measurements and taking the median of each sample. Therefore, sample aliquots were diluted with solvent (20 % MeOH) prior to measurements. A pronounced peak was visible in our spectra at λ=338 nm. We then compared these spectra against a purpurogallin standard (CarboSynth, UK), and although the spectral signatures were not perfectly aligned, we still attribute this dominant peak to the presence of intracellular purpurogallin-derivatives for several reasons: (i) Remias et al. (2012b) and Prochazkova et al. (2021) identified purpurogallin as the dominant phenol for *A. alaskana* and *A. nordenskiöeldii*, respectively (Remias et al., 2012) (ii) the peak shape and λ_{max} looked like the one shown in the Supplemenetary Material of Remias et al. (2012b) (iii) the peak shape and shoulders resemble the one of the purpurogallin standard (brown pattern in Figure 1) (iv) we identified a bathochromatic effect increasing λ_{max} once algal matrix was not containing purpurogallin (grey line in Figure S1) was spiked

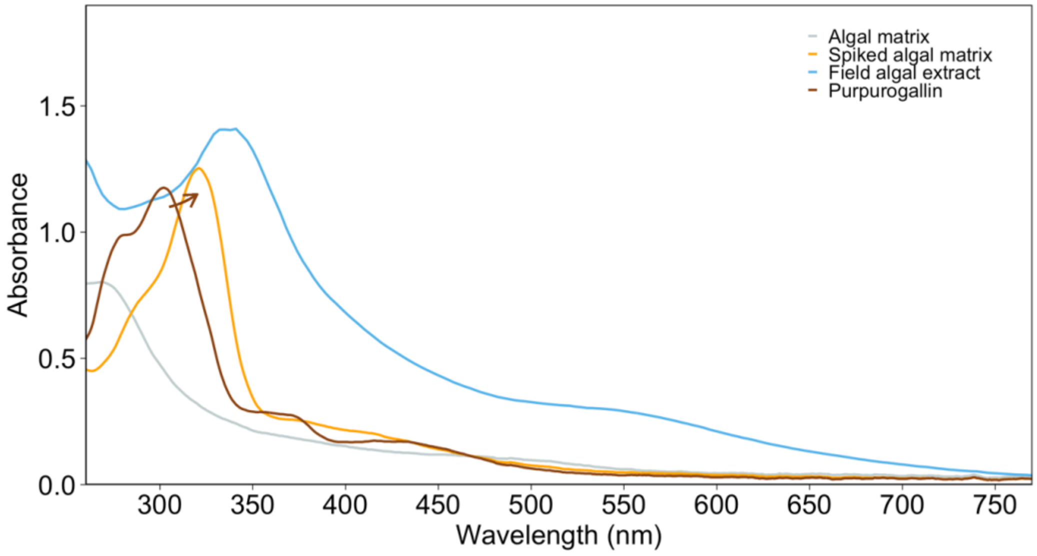

with a purpurogallin standard (orange line in Figure S1). Subsequently, the purpurogallin concentration in the hydrophilic field sample extracts (blue pattern in Figure S1) was obtained by performing a 4-point calibration against the standard aligning the absorbance peaks. Using this set-up, the detection limit for purpurogallin was 0.7 mg l^{-1} (in all cases concentration values of samples were above the limit of detection).

Figure S1. Spectral shift in absorption of purpurogallin due to a matrix effect: brown line shows the purpurogallin standard (20% MeOH), grey line the un-spiked algal extract from a snow algal dominated population not containing purpurogallin, orange line the algal extract spiked with purpurogallin standard revealing a bathochromatic shift of λmax towards the VIS wavelength range and the blue line showing the hydrophilic extract after phase separation of a sample from a glacier ice algal dominated site (MIT5).

Identification of purpurogallin with high resolution LC-HRMS/MS

Using an ultra-high performance liquid chromatography system hyphenated with an Orbitrap high-resolution tandem mass spectrometer, UHPLC-MS (Dionex Ultimate 3000 NCS-3500RS Nano Proflow system and Q Exactive HF, Thermo Scientific, Germany), we confirmed the presence of both purpurogallin-carboxylic-acid and purpurogallin-carboxylic-acid-glycopyranoside in hydrophilic algal cell extracts (see pigment methods) and dissolved fraction of glacier ice (exometabolome, melted glacier ice filtered through 0.2 µm PES filter). Purpurogallin-carboxylic-acid-glycopyranoside was present in higher quantities within the algal extracts matching the observations by Remias et al. (2012b).

The dissolved fraction (no extraction required) and the intracellular algal pigment extract were analysed in positive and in full scan mode at 240,000 in mass resolution at m/z 200. Five µl were loaded onto a C18 trap column (100 Å, C18, 0.3 mm x 5 mm, nanoViper, Thermo Scientific) by full-loop injection at a flow of 30 µL min^{-1} loading solvent C (0.1% formic acid) for 2 minutes. Subsequently, analytes were eluted onto a nanoflow UHPLC analytical column (PepMap RSLC, C18, 2 µm, 100 Å, 75 µm x 25 cm, Thermo Scientific). The mobile phase for chromatographic separation consisted of solvent B (0.1 % formic acid in acetonitrile) and solvent A (0.1 % formic acid in water) which were used in a linear gradient spanning in total a 30 minute run time. During the analysis UHPLC-MS grade solvents were used at any time.

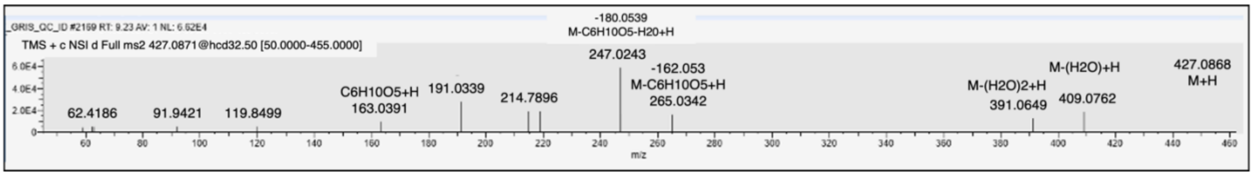

Figure S2. MS2 fragmentation pattern of purpurogallin carboxylic acid-6-O-b-D-glucopyranoside (C18H18O12), 426.0668 MW in positive ionisation mode.

Dissolved organic carbon sampling and measurements

Samples for dissolved organic carbon (DOC) were filtered through 0.3 µm filters (GF/F Whatman) and collected into pre-combusted glass vials (450 °C) followed by acidification with HCL to a pH of 2. Samples were stored at 4°C in the dark until analysed on a total organic carbon analyser (TOC, Shimadzu TOC-V, 3% precision). The average DOC concentrations were used to build the absorption coefficient of chromophoric dissolved organic matter.

Algal absorption coefficients

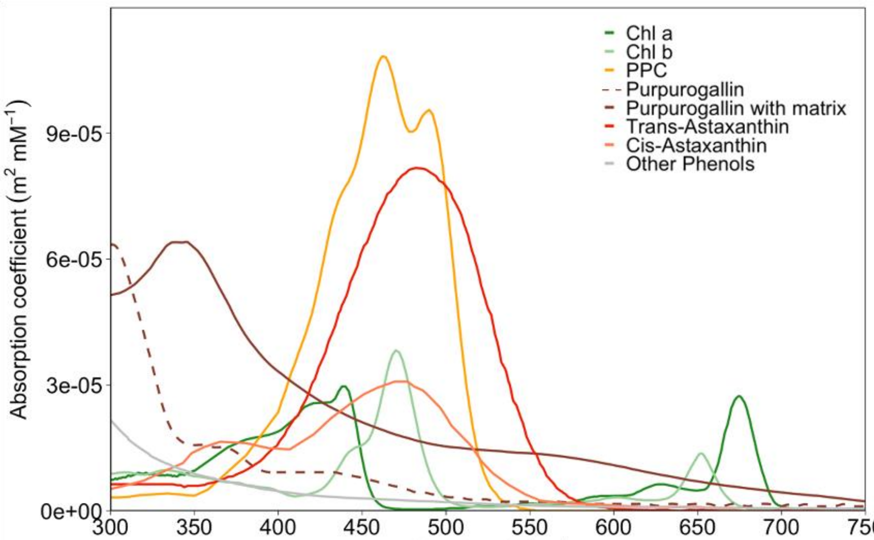

Figure S3. Molar and mass-specific absorption coefficients of different pigments. Coefficients for Purpurogallin and Purpurogallin with matrix and other phenols derived in this study, while the other ones were derived from other studies (see method description in main text). Note that the coefficient of *trans*-astaxanthin was extrapolated between 300 to 350 nm.

To illustrate the role of individual pigments for absorption of glacier ice and snow algae, we calculated their average pigment concentrations (mM): (i) for snow algae we used the average pigment pool composition from the snow sites MIT1+2, where glacier ice algae biomass and pigment contribution (purpurogallin) was neglectable and; (ii) for glacier ice algae we calculated average pigment compositions from all bare ice sites from which the snow algal pigment pool (e.g. average Chl *a* concentration per biomass for snow algae) was subtracted depending on the snow algal biomass found at the specific sites. Purpurogallin was directly normalised to glacier ice algae biomass.

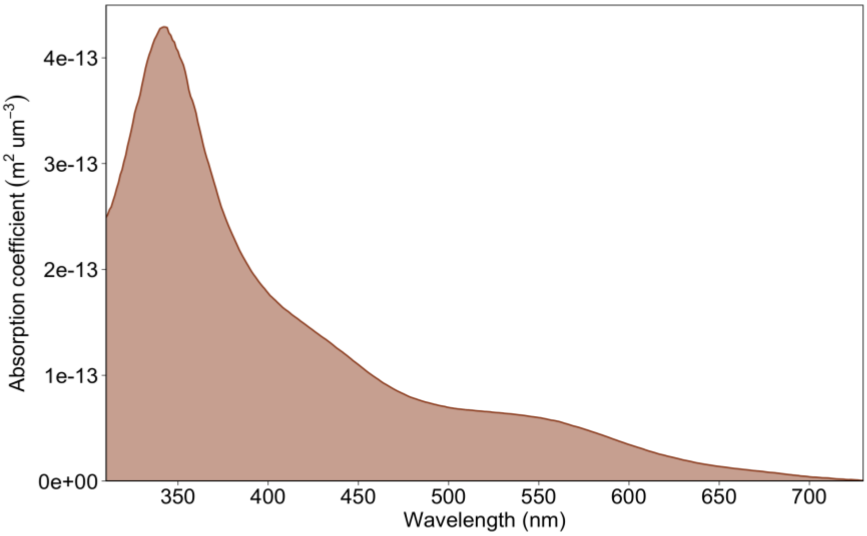

Figure S4. Glacier ice algal absorption spectra derived from whole-cell absorption measurements on filters. Note that shape resembles the absorption of extracted purpurogallin and pigment leakage upon thawing and wetting of the filter surface may have impacted this spectrum.

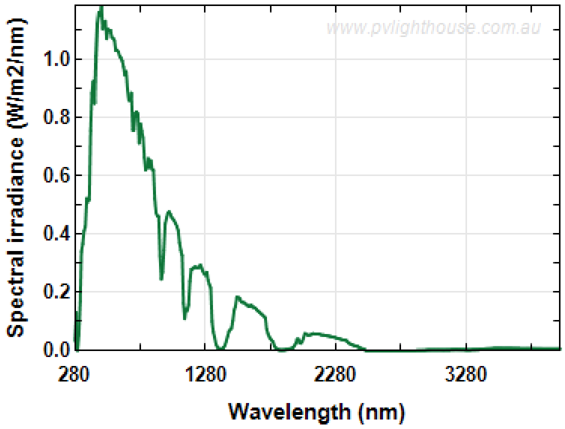

Figure S5. Spectral irradiance derived for PVSystems solar irradiance program (https://pvlighthouse.com.au, last access: February 2022) for 60◦ N and 24 July 2019 12 pm midday (in W m2 nm-1 λ280−750nm) and using the default settings i.e. the global irradiance (being the sum of direct and diffuse irradiance) and horizontal plane (perpendicular to the direction of sunlight).

Supplementary results

Table S4. Average algal abundances in cells ml^{-1} (SA=Snow algae, A.N.=*Ancylonema nordenskiöldii*, A.A.=*Ancylonema alaskanum*) and cell volumes (µm^{-3}) with SD= Standard deviations. The three last rows display the averages for the different regions.

Site	Abundance								Cell volumes					
	Total	± SD	A.A.	± SD	A.N.	± SD	SA	± SD	SA	± SD	A.A.	± SD	A.N.	± SD
BR	33188	13937	28725	12331	1575	1757	2888	1342	7328	1921	1796	313	1651	
HE1	27600	10881	25950	9828	1200	1239	450	195	7096	2749	1147	329	1967	885
HE2	5063	5291	2325	1536	2738	4079	na		1821		930		1888	
MIT1	1163	620	788	684	na		375	130	4200	408	na		na	
MIT2	7500	1897	1988	1375	na		5513	893	3728	941	na		na	
MIT3	29063	6574	9450	3820	17663	4117	1950	1081	7130	4719	790	214	2530	187
MIT4	33600	20112	18563	5885	12938	14435	2100	640	15116	5273	654	83	2499	775
MIT5	57525	16730	24863	2190	29513	16732	3150	1622	20101	5853	553	102	2372	300
MIT6	55000		23000		32000		0		na		1291		1829	
HE1+2	16331	8086	14138	5682	1969	2659	450	195	4459	2749	1039	329	1928	885
MIT1+2	4331	1258	1388	1030			2944	511	3964	675				
MIT3-6	43797	14472	18969	3965	23028	11761	1800	1114	12197	5282	822	133	2307	421

Table S5. Pigment composition in % of total pigment pool used in Figure 4. Chl= Chlorophylls, PC= photoprotective carotenoids, PPG =Purpurogallin. The three last rows display the averages for the different regions.

Station	Chl	PC	Astaxanthins	PPG
BR	0.58	0.30	87.44	11.68
HE1	0.63	0.81	35.30	63.26
HE2	1.51	1.12	33.10	64.27
MIT1	1.85	0.28	97.87	0.00
MIT2	1.01	0.64	98.35	0.00
MIT3	1.09	0.91	55.92	42.08
MIT4	1.35	2.08	76.75	19.82
MIT5	1.74	1.17	65.83	31.26
MIT6	0.44	0.24	63.10	36.21
HE1+2	1.07	0.97	34.2	63.77
MIT1+2	1.43	0.46	98.11	
MIT3-6	1.16	1.1	65.4	32.34

Table S6. Average pigment ratios to Chl *a* (mg L^{-1} concentrations) followed by the standard deviation (corresponding Table 1 in mM in main manuscript).

Site	Chl *a*		Pheo:Chl a		Neo:Chl a		Vio:Chl a		Lut:Chl a		Zea:Chl a		Zea:Vio	PPG:Chl a		Trans-Asta:Chl a		Cis-Asta-ester:Chl a		Trans-Asta-ester:Chla	
BR	0.129	±0.061	0.48	±0.83	0.21	±0.16	0.11	±0	0	±0	0.04	±0.07	0.13	9.27	±4.61	0.72	±0.15	87.7	±8.72	32.3	±5.77
HE1	0.004	±0.003	0	±0	0.25	±0.14	0.37	±0	0	±0	0.05	±0.04	0.18	126	±177.67	0.14	±0.01	9.09	±5.12	18.1	±4.89
HE2	0.004	±0.005	0	±0	0.08	±0.04	0.13	±0.12	0.07	±0	<DL	±0.01	0.08	74.1	±101.14	0.02	±0.03	11.9	±8.61	16	±7.18
MIT1	0.004	±0.003	0	±0	<DL	±0	<DL	±0.09	0.12	±0	<DL	±0		<DL	±0	0.02	±0.02	34.7	±12.76	11.7	±2.34
MIT2	0.034	±0.028	1.16	±0.49	0.08	±0.05	0.08	±0.16	0.13	±0	0.03	±0.05	0.53	<DL	±0	0.04	±0.02	84.7	±30.51	17.4	±7.7
MIT3	0.051	±0.039	0.53	±0.35	0.07	±0.02	0.09	±0.11	0.42	±0	0.05	±0.04	0.71	30.4	±22.93	0.21	±0.19	21.4	±2.46	22	±4.16
MIT4	0.047	±0.013	0.41	±0.29	0.3	±0.13	0.39	±0.25	0.63	±0	0.09	±0.16	0.21	11.9	±14.5	0.91	±0.41	27.2	±7.11	28.2	±7.07
MIT5	0.081	±0.024	0.26	±0.07	0.1	±0.14	0.13	±0.1	0.35	±0	0.09	±0.08	0.84	12.8	±13.13	0.64	±0.27	27.6	±5.99	24.7	±1.8
MIT6	0.008	±0.008	0.9		0.03		0.12		0.2		0.05		1.78	51.6		0.04		33		123	

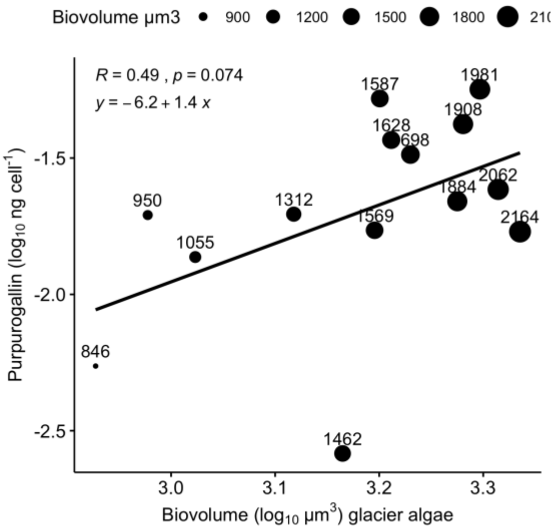

Figure S6. Correlation between the log-transformed purpurogallin concentrations and total biovolume (product of cell numbers and cell volume) of both *A. alaskanum* and *A. nordenskiöldii*. Point sizes and point labels reflect cell volumes.

Table S7. $CDOM_{a375}$ concentrations for the different sampling sites.

Region	a375 (m^{-1})
BR	1.08
HE1	0.38
HE2	0.87
MIT1	0.02
MIT2	0.13
MIT3	1.32
MIT4	1.53
MIT5	2.02
MIT6	0.66
HE1+2	0.63
MIT1+2	0.08
MIT3-6	1.38

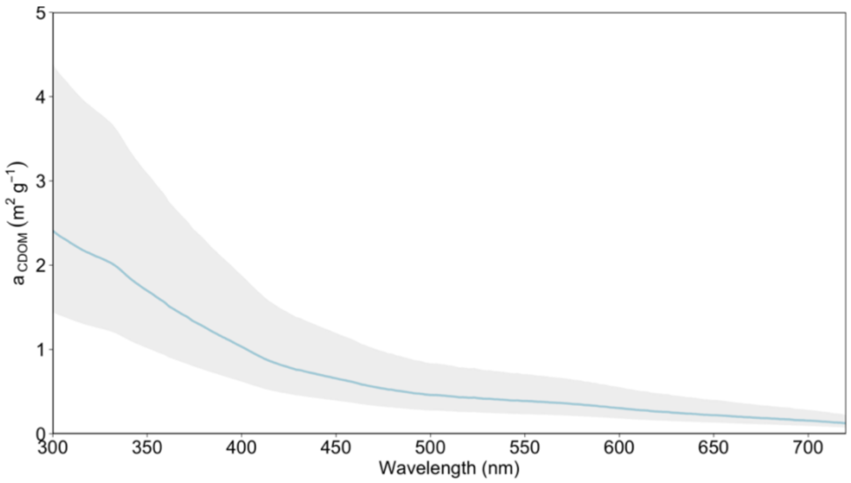

Figure S7. Average CDOM absorption coefficient derived from the bare ice sampling sites on Mittivakkat. The shaded area depicts the variation in absorption coefficient based on the minimum and maximum DOC concentrations.

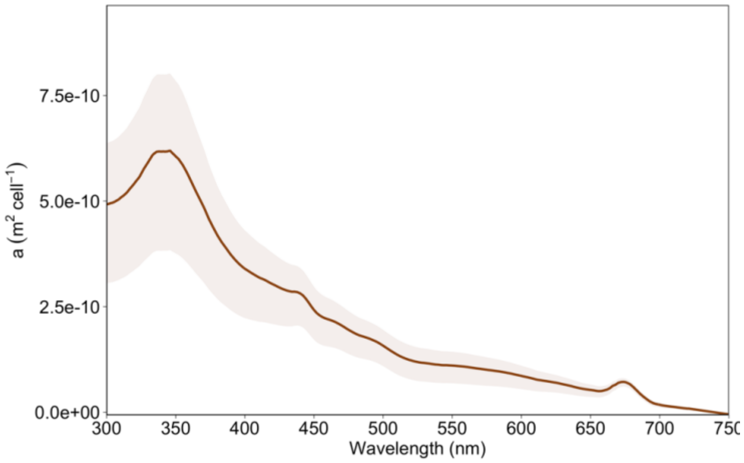

Figure S8. Variation in cellular absorption coefficient due to variation in purpurogallin per cell (for the average and upper and lower limit of the 95% CI). The amount of all other pigments was kept constant.

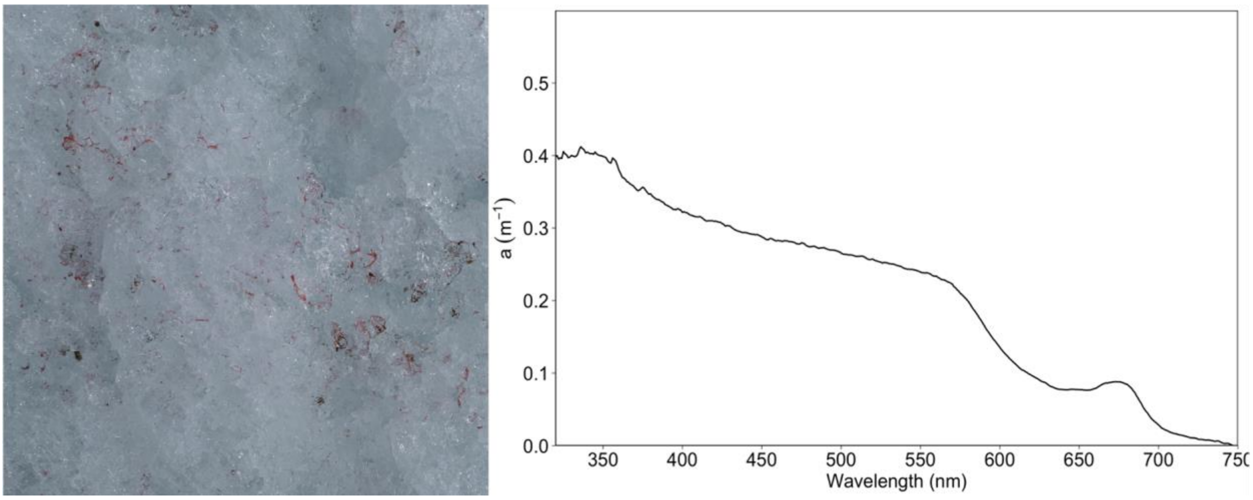

Figure S9. Red-coloured bare ice on Mittivakkat, close to MIT5. Absorption spectrum of the red coloured bare ice spot shows snow algal absorption signature and now purpurogallin absorption peak, which was visible on other bare ice areas.

Supplementary references

Aigner, S., Remias, D., Karsten, U., and Holzinger, A. (2013). Unusual phenolic compounds contribute to ecophysiological performance in the purple-colored green alga Zygogonium ericetorum (Zygnematophyceae, Streptophyta) from a high-alpine habitat. *J. Phycol.* 49, 648–660. doi:10.1111/jpy.12075.

Bidigare, R. R., Ondrusek, M. E., Ii, M. C. K., Iturriaga, R., Harvey, H. R., and Macko, S. a (1993). Evidence for a photoprotective function for secondary carotenoids of snow algae taxonomy, life histories , ecology, and geographical habitats and polar regions. 434, 427–434.

Bohn, N., Painter, T. H., Thompson, D. R., Carmon, N., Susiluoto, J., Turmon, M. J., et al. (2021). Optimal estimation of snow and ice surface parameters from imaging spectroscopy measurements. *Remote Sens. Environ.* 264, 112613. doi:10.1016/j.rse.2021.112613.

Cook, J. M., Tedstone, A. J., Williamson, C., McCutcheon, J., Hodson, A. J., Dayal, A., et al. (2020). Glacier algae accelerate melt rates on the south-western Greenland Ice Sheet. *Cryosphere* 14, 309–330. doi:10.5194/tc-14-309-2020.

Dial, R. J., Ganey, G. Q., and Skiles, S. M. (2018). What color should glacier algae be ? An ecological role for red carbon in the cryosphere. *FEMS*, 1–9. doi:10.1093/femsec/fiy007.

Greg, S., and Malkin, S. Y. (2015). Package "phytotools" Type Package Title Phytoplankton Production Tools.

Holzinger, A., Albert, A., Aigner, S., Uhl, J., Schmitt-Kopplin, P., Trumhová, K., et al. (2018). Arctic, Antarctic, and temperate green algae Zygnema spp. under UV-B stress: vegetative cells perform better than pre-akinetes. *Protoplasma* 255, 1239–1252. doi:10.1007/s00709-018-1225-1.

Jassby, A. D., and Platt, T. (1976). Mathematical formulation of the relationship between photosynthesis and light for phytoplankton. *Limnol. Oceanogr.* 21, 540–547. doi:10.4319/lo.1976.21.4.0540.

Remias, D., Schwaiger, S., Aigner, S., Leya, T., Stuppner, H., and Lütz, C. (2012). Characterization of an UV- and VIS-absorbing, purpurogallin-derived secondary pigment new to algae and highly abundant in Mesotaenium berggrenii (Zygnematophyceae, Chlorophyta), an extremophyte living on glaciers. *FEMS Microbiol. Ecol.* 79, 638–648. doi:10.1111/j.1574-6941.2011.01245.x.

Williamson, C. J., Cook, J., Tedstone, A., Yallop, M., McCutcheon, J., Poniecka, E., et al. (2020). Algal photophysiology drives darkening and melt of the Greenland Ice Sheet. *Proc. Natl. Acad. Sci.*, 201918412. doi:10.1073/pnas.1918412117.

Wright, S. W. (1991). Improved HPLC method for the analysis of chlorophylls and carotenoids from marine phytoplankton. *Mar. Ecol. Prog. Ser.* 77, 183–196. doi:10.3354/meps077183.
